# Colon cancer awareness for prevention: Call for global initiatives

**DOI:** 10.4103/1477-3163.48607

**Published:** 2009-03-06

**Authors:** Gopala Kovvali

**Affiliations:** Department of Genetics, Rutgers University, 145 Bevier Road, Piscataway, NJ 08854-8082, Carcinogenesis Foundation, 22 Heritage Drive, Edison, NJ, 08820, USA E-mail: gopala@carcinogenesis.com; Kovvali@dls.rutgers.edu

March has been recognized as National Colon Cancer Awareness Month in the United States of America, by a resolution of the US Senate in 1999. The goals of this initiative are to generate widespread awareness about colorectal cancer and encourage people to learn more about prevention of the disease through regular screening and a healthy lifestyle.

Every year, more than 940,000 cases of colon cancer occur worldwide and nearly 500,000 people die from it. It is the second leading cause of cancer death among men and women in the US. Colorectal cancers are the most preventable of all cancers. Early detection of precancerous lesions is the key for prevention.

The burden of cancer doubled between 1975 and 2000, and it is predicted to double again by 2020 and triple by 2030. Sadly, the burden is shifting to the developing or less developed nations. According to the 2008 World Cancer Report published by the World Health Organization, the total number of cases of cancer in the developing world between 2000 and 2020 is expected to increase by 73 per cent.

Why is this increased risk of cancer incidence with epidemic proportions in developing countries happening? A few years ago, I wrote an editorial in this journal and proposed a concept “Acquired Risk for Cancer Incidence”, abbreviated as ARCI. The concept I proposed was based on empirical and semi-empirical data which suggested that individuals who migrated to western countries acquire an increased risk of cancer incidence, akin to the risks known to the population of the country they migrated to. [New paradigms, new Hopes: the need for socially responsible research on carcinogenesis. J Carcinog 2005, 4:22]. It is interesting that the report by the WHO suggests that changes in lifestyle similar to the ones in the western nations could confer additional risk of cancer incidence, which offers a basis for investigating the phenomenon of ARCI.

In developing countries, more and more people are embracing western lifestyles, including smoking, high-fat diets, fast food and less physical activity. As developing countries become urbanized, patterns of cancer, including those most strongly associated with diet, tend to shift towards those of economically developed countries.

Populations in these countries are expected to grow by 38 percent by 2030. And, these countries will have a high number of older people as populations age, increasing the number of cancers in these countries. While the statistics presented here are of serious concern, there are opportunities to make a difference.

The Carcinogenesis Foundation calls for declaration of March as a global colon cancer awareness month by the World Health Organization. Let us initiate a global carcinoprevention drive.

